# Therapeutics

**Published:** 1877-11

**Authors:** 


### IV. Therapeutics.

Croton-chloral.—*La France Médicale*, July 25, 1877.—Dr. Livon, in a recent study of this drug, at the physiological laboratory at Marseilles, arrives at the following conclusions:

This remedy is destined in the hands of physicians, to render as great service as chloral has done. The author does not regard it as a sovereign remedy as it may fail in certain cases, but it relieves pain, and in less doses than chloral, 10, 20, or 30 centigrammes being sufficient to soothe neuralgic pains without profound and forced sleep. Liebreich had already noticed this special action upon the sensibility of the head, but he regards it as perfectly innocuous to the stomach and other organs, while Livon recognizes its irritating and caustic properties, and says it is best not to give it by the mouth in inflammatory states of the digestive system. In patients who have a tendency to cerebral congestion, it is best to abstain from the use of croton-chloral. In case of accidents, artificial respiration should be used, and electricity along the spine and over the course of the pneumogastric.

It is not dangerous to the heart except in large doses. The difference between its action and that of chloral is such that Liebreich advises its use instead of chloral in subjects with cardiac disease.

From all his observations and autopsies, Livon draws the following conclusions:

1. Croton-chloral acts upon the central nervous system.
2. In small doses it acts upon the brain alone, and by its intermediation solely upon the sensitive cranial nerves.
3. In larger doses, its action extends to the cord and sensitive spinal nerves.
4. The motor nerves are only acted upon secondarily.
5. It is only in exaggerated doses that the arrest of the heart's action, and of respiration, may be provoked by cessation of nervous afflux.

It may be given by the stomach or hypodermically. The author's formula for administration by the stomach is:

| | | |
|---|---|---|
| Croton-chloral, | - - - | 2 grammes |
| Glycerine (warm) | - - | 6 " |
| Ext. licorice | - - - | 4 " |
| Water } Syrup } | aa - - - | 45 " |

For hypodermic injection his formula is:

| | | |
|---|---|---|
| Croton-chloral | - - - | 1 gr. 60 c. |
| Glycerine } Cherry laurel water } | aa. - | 16 grammes |

Each gramme of the solution contains 5 centigrammes.

Divided doses of 5, 10, or 20 centigrammes repeated as required several times in succession, generally succeed in quieting pain. From 50 centigrammes to 1 gramme instantly relieve pains of considerable intensity, and for very severe pain, the dose may be carried up to 3, 4 or even more grammes at once.

The author does not recommend intra-venous injections, on account of the difficulties and dangers that may follow.

For physiological experiments, however, intra-venous injections of croton-chloral made with care, at a certain distance from the heart, constitute a precious means for immobilizing animals destined for the most delicate and difficult vivisections.

L. W. C.

Croton-chloral for Ciliary Neuralgia.— Friedinger.— (*Wiener Med. Wochenschrift*, No. 31, 1877.) Croton-chloral seems to have an almost specific influence on the sensory fibres of the fifth nerve, and it can be more surely relied upon to allay those fearful pains, which attend the violent inflammations of the iris and choroid, and are known as ciliary neuralgia. In all cases in which it was given for this neuralgia, it has exerted its anæsthetising effect without producing any collateral disturbance. This is the formula:

| | | | |
|---|---|---|---|
| ℞ Croton-chloral hydrate | - | 1 gramme | |
| Spir. vin. rectif. | - - - | 4 " | |
| Aq. destill. | - - - | 150 " | |
| Syr. aurant. cort. | - - | 15 " | M. |

1 tablespoonful every two hours.

